# Corticosteroids for Posttransplant Immune Reconstitution Syndrome in *Cryptococcus gattii* Meningoencephalitis: Case Report and Literature Review

**DOI:** 10.1093/ofid/ofz460

**Published:** 2019-10-23

**Authors:** Gregory S Canfield, Andrés F Henao-Martínez, Carlos Franco-Paredes, Kristen Zhelnin, Michael L Wilson, Katherine C Shihadeh, David Wyles, Edward M Gardner

**Affiliations:** 1 Department of Infectious Diseases, University of Colorado School of Medicine, Aurora, Colorado, USA; 2 Hospital Infantil de Mexico, Federico Gomez, Mexico City, Mexico; 3 Deparment of Pathology, Denver Health Medical Center, Denver, Colorado, USA; 4 Department of Pharmacy, Denver Health Medical Center, Denver, Colorado, USA; 5 Department of Infectious Diseases, Denver Health Medical Center, Denver, Colorado, USA

**Keywords:** corticosteroids, cryptococcus gattii, immune reconstitution syndrome, meningoencephalitis, transplant

## Abstract

*Cryptococcus gattii* represents an emerging fungal pathogen of immunocompromised and immunocompetent hosts in the United States. To our knowledge, this is the first case of posttransplant immune reconstitution syndrome due to *C. gattii* meningoencephalitis successfully treated with corticosteroids. We also report successful maintenance phase treatment with isavuconazole, a novel triazole, following fluconazole-induced prolonged QT syndrome.

A 55-year-old male with history of renal transplantation 9 years earlier presented to the hospital with confusion, headaches, neck pain, cough, nausea, and vomiting. The patient, now disabled, previously was an airline mechanic who had travelled remotely to China, the Philippines, Hong Kong, and Surinam. No significant changes to his immunosuppressive regimen were noted in the months prior to admission (Figure 1A).

**Figure 1.**
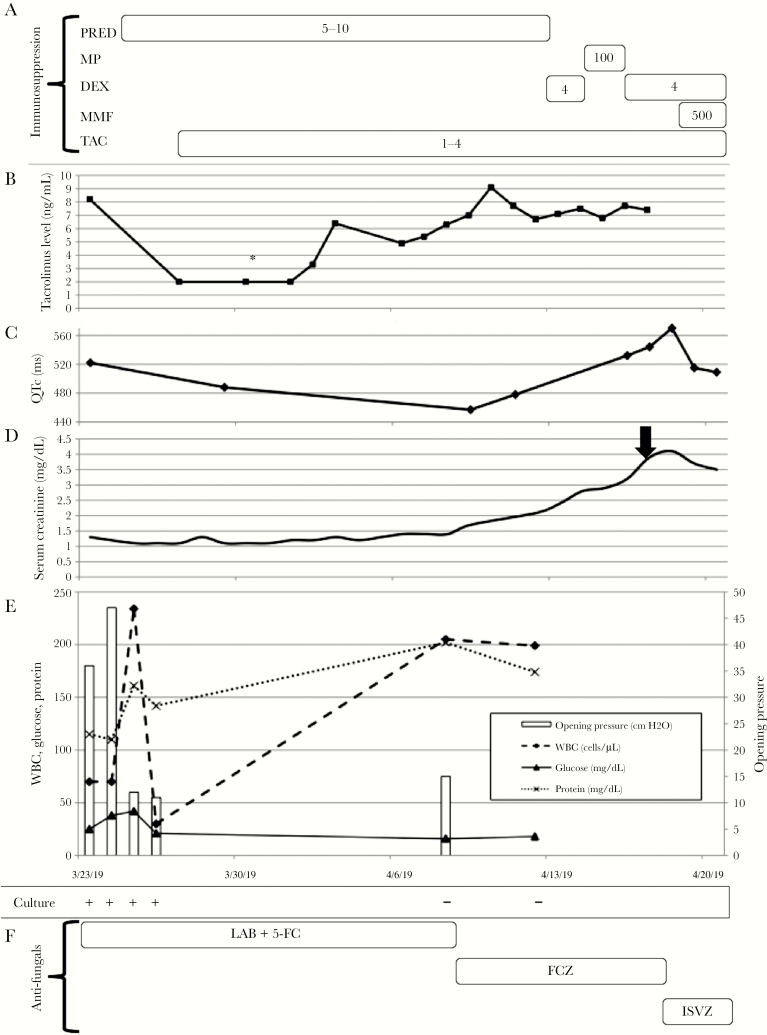
Timeline of (A) immunosuppression dosing, (B) tacrolimus level, (C) electrocardiogram corrected QT interval length (QTc) in milliseconds (ms), (D) serum creatinine, (E) opening pressure and cerebrospinal fluid (CSF) results, and (F) CSF culture and antifungal therapy. A, immunosuppression regimen: numbers inside of boxes represent daily dose in milligrams (mg) of an immunosuppressive agent. Patient’s outpatient immunosuppression regimen prior to admission consisted of tacrolimus 1mg in the morning and 2mg in the evening, mycophenolate 1000mg twice daily, and prednisone 5mg daily. B, tacrolimus trough level: each point represents an individual day of monitoring. The asterisk indicates tacrolimus dose less than equal to 2 mg/dL (the lowest level of detection of the test) and may range anywhere from 0 to 2 mg/dL. C, QTc interval in ms: each point represents an individual electrocardiogram. D, serum creatinine with black arrow indicating date of detection of *Klebsiella pneumonia* and vancomycin-resistant *Enterococcusfaecium* urinary tract infection. E, CSF white blood count, glucose, protein, opening pressure, and culture results: plus signs (+) indicate positive CSF culture and minus signs (-) indicate negative CSF culture. Each individual marker, bar graph, and plus or minus sign represents an individual data point per day. The X-axis in Figure 1E (Time) corresponds to tick marks in each individual section. F) Antifungal treatment regimen. Abbreviations: DEX, dexamethasone; FCZ, fluconazole; ISVZ, isavuconazole; LAB, liposomal amphotericin B; MMF, mycophenolate; MP, methylprednisolone; PRED, prednisone; TAC, tacrolimus; 5-FC, 5-flucytosine.

Admission laboratory tests showed a white blood cell count (WBC) of 7.2 x 10^6^/mL, creatinine of 1.3 mg/dL (baseline creatinine 1.2 mg/dL), and non-reactive fourth generation HIV antigen/antibody test. A lumbar puncture performed upon admission showed a cerebrospinal fluid (CSF) opening pressure (OP) of 36 cm H_2_O (normal < 20 cm H_2_O), WBC of 70 cells/μL (62% lymphocytes), glucose 25 mg/dL, and protein 115 mg/dL; CSF India ink stain was positive for yeast forms, meningoencephalitis CSF polymerase chain reaction (Biofire FilmArray, Salt Lake City, UT) was positive for *Cryptococcus neoformans/gattii*, and culture yielded *Cryptococcus gattii*. A cryptococcal antigen was reactive at a titer of 1:2560 in serum and CSF. Dilated fundoscopic examination was negative for signs of cryptococcal intraocular invasion. Computed tomography (CT) scan of the chest demonstrated 2 solid nodules in the right lung (Supplementary Figure 1A). Biopsy of the lower lobe nodule demonstrated encapsulated yeast forms consistent with *Cryptococcus* (Supplementary Figure 1B–D).

He received liposomal amphotericin B and flucytosine as induction therapy. Tacrolimus was restarted after day 5 of his hospitalization (Figure 1A). Given persistent confusion on day 5 of his hospitalization, magnetic resonance imaging (MRI) of the brain revealed multiple acute ischemic infarcts in the bilateral cerebellar hemispheres. Transthoracic echocardiogram with contrast demonstrated a thrombus within the apex of the left ventricle. By day 9 of his hospitalization, his mental status was back to baseline and he was fully conversant. After 2 weeks of induction therapy, his CSF cultures were negative and CSF OP normalized, albeit with persistent CSF pleocytosis (Figure 1E). The patient was transitioned to fluconazole consolidative therapy per published guidelines [1].

Despite initial improvement, he developed progressive deterioration of his mental status and recurrence of his nausea and vomiting. These clinical changes prompted repeat lumbar puncture that showed persistent negative CSF culture, 205 WBC/µL (95% lymphocytes), and an OP of 15 cm H_2_O in his CSF (Figure 1E). Although a serum CD4 T cell count was not obtained, a ~2.5 fold rise in the serum absolute lymphocyte count (ALC) from admission (ALC = 500 cells/µL; normal 900–4000 cells/µL) to day 13 of induction therapy (ALC = 1290 cells/µL) was observed. Noncontrast CT of the brain did not reveal new hemorrhagic or ischemic stroke. Minimum inhibitory concentration (MIC) of 2 µg/mL to fluconazole of his initial *C. gattii* isolate suggested antifungal efficacy based upon prior epidemiologic cutoff values [2, 3]. Dexamethasone 4mg daily was started for presumed immune reconstitution syndrome (IRS); he remained on tacrolimus. His course was complicated further by prolonged QTc (570ms; Figure 1C), which necessitated a switch in therapy to isavuconazole, a novel triazole that shortens the QT interval [4]. Transition from dexamethasone 4mg daily to methylprednisone 125 mg every 6 hours for 3 days was advised due to presumed allograft rejection. Following high dose methylprednisolone, he was transitioned back to dexamethasone and completed a taper as follows: 2 weeks of dexamethasone 4mg daily, followed by 1 week dexamethasone 3mg daily, followed by 3 days dexamethasone 2mg daily, and followed by 3 days of dexamethasone 1mg daily. After 3 days of corticosteroids and isavuconazole the patient’s mental status and QTc improved. Recurrence of nausea and vomiting 1 week after completion of corticosteroids prompted repeat MRI brain, which demonstrated a new lacunar infarct of the left internal capsule and new enhancement of bilateral basal ganglia encompassing dilated perivascular spaces along with previously demonstrated changes (Supplementary Figure 2). His symptoms improved with antiemetics without need of repeat corticosteroid burst. Further measures of QTc interval at 1 (495ms) and 2 (487ms) months postisavuconazole initiation demonstrated durable QTc shortening effect. The patient was discharged on isavuconazole, cyclosporine, and low dose prednisone.

## RESULTS 

*C. gattii* represents an emerging fungal pathogen that inhabits eucalyptus-type flora, soil, and bird droppings in tropical areas. A North American outbreak of *C. gattii* was observed in the Pacific Northwest in 2004 [5]. *C. gattii* infection (versus *C. neoformans*) was distinguished by a higher MIC to fluconazole and its ability to promote aggressive CNS infection [2, 3]. It has become clear that chronic medical issues (ie, uncontrolled diabetes mellitus or end-stage renal disease) and macrophage dysfunction associated with anti-granulocyte–macrophage colony-stimulating factor autoantibodies are risk factors for *C. gattii* infection in immunocompetent hosts [6]. Multilocus sequence typing showed that 92% of Pacific Northwest isolates belonged to VGII subtype, while those found in the southeastern and southwestern United States comprised VGI and VGIII subtypes [7–10]. Although a subtype was not ascertained in our patient, we suspect a non-VGII sporadic isolate of *C. gattii* given the low fluconazole MIC and absence of travel to the Pacific Northwest. He likely acquired *C. gattii* either domestically or during international travel earlier in his life. Prior global surveys show VGI and VGIII subtypes predominate in Asia and North America (except the Pacific Northwest), respectively [11].

We suspect reactivation of latent *C. gattii* infection caused his disseminated infection. *C. gattii* infection in solid organ transplant (SOT) recipients shows smaller nodular or interstitial pulmonary infiltrates, more frequent leptomeningeal enhancement, and higher rates of intensive care unit admission and mortality (70%) [12]. Conversely, the risk of death was found to be lower amongst US SOT recipients with cryptococcal infection but not HIV [13], a result that may be explained by the low prevalence of C. gattii infection in this cohort (ie, 5 of 145 cryptococcosis cases). Lacunar stroke is a frequent complication of delayed cryptococcal meningitis diagnosis [14].

Immune reconstitution syndrome represents a paradoxical worsening of signs and symptoms after an initial response (despite effective therapy and microbiologic cure) and cannot be explained by a persistent primary infection, new secondary infection, or adverse drug events. Posttransplant IRS following cryptococcosis is seen in 5%–11% of patients approximately 4 weeks to 9 months after initiation of antifungal therapy [15]. Manifestations of IRS in SOT recipients include lymphadenitis, cellulitis, pulmonary nodules, aseptic meningitis, or cerebral mass lesions. Central nervous system disease and discontinuation of calcineurin inhibitor therapy were associated independently to increase the risk of IRS by 5–6 fold in SOT recipients with cryptococcosis [16]. Tacrolimus exerts its antifungal activity through inhibition of phosphatase function of the calcineurin homolog expressed in *C. neoformans* and by limiting the interaction of calcineurin binding protein 1 (CBP1) and calcineurin [17, 18]. Risk factors for IRS following *C. gattii* infection include female sex, CNS disease, and higher median CD4 counts [19]. In our patient, the development of recurrent encephalopathy after initial improvement on antifungal therapy, persistence of aseptic meningitis despite effective induction therapy and control of CSF OP, discontinuation of tacrolimus on admission, an antecedent rise of the serum absolute lymphocyte count prior to IRS diagnosis, and the clinical response to prolonged corticosteroid taper argue in favor of *C. gattii*-induced posttransplant IRS.

The immunologic mechanisms mediating posttransplant IRS are obscure. It is suggested that the reduction of immunosuppression and administration of antifungal therapy favor a shift from an anti-inflammatory Th2/Treg-mediated cytokine response to a proinflammatory Th1/Th17-mediated response. It is this overzealous reversion to a proinflammatory response that potentially promotes IRS in the wake of cryptococcal clearance. Prevalence estimates of IRS during *C. gattii* infection are currently limited to case series and reports [20–24]. Interestingly, in a small immunocompetent cohort of patients presenting with severe cryptococcal CNS infection and post infectious inflammatory response syndrome (PIIRS), a skewing towards a nonprotective M2 macrophage response was observed [25]. Whether this M2 macrophage response contributes to posttransplant IRS following cryptococcal meningitis is unknown.

In patients diagnosed with IRS, corticosteroids are employed to tip the net state of immunosuppression towards an anti-inflammatory response. Precedence for the treatment of posttransplant IRS in cryptococcosis with corticosteroids is limited to case reports [26–29]. Aside from the SOT recipient population, corticosteroids also have shown promise for the treatment of IRS in HIV-associated *C. neoformans* meningoencephalitis [1]. Corticosteroids also were shown to provide benefit in the salvage treatment of PIIRS in immunocompetent patients with cryptococcal meningitis [25]. Infectious Disease Society of America guidelines caution against the use corticosteroids prior to proof of culture sterilization following induction therapy. This treatment approach is supported by the increased mortality observed among those receiving corticosteroids as an adjunct to induction therapy (versus induction therapy alone) for the treatment of HIV-associated cryptococcal meningitis [30]. In our patient, sterilization of the CSF was demonstrated by day 16 of induction therapy, which validated our diagnosis of IRS and treatment with corticosteroids.

His course was complicated further by the development of prolonged increase in the QTc interval, which correlated with the start of fluconazole (Figure 1C and 1E). The patient was transitioned to isavuconazole and experienced a durable reduction in his QTc (Figure 1C). Unfortunately, an isavuconazole MIC of the initial *C. gattii* isolate was not obtained; although, to date, our patient has experienced a successful clinical response to this agent. The therapeutic efficacy of isavuconazole in treating CNS cryptococcosis is limited. The VITAL trial showed a successful response to isavuconazole in 6 out of 9 patients treated for cryptococcosis [31]. Three of the 9 patients presented with *C. gattii* associated CNS infection (none with SOT), and all with successful responses. Prior in vitro susceptibility data indicates low epidemiological cutoff values to isavuconazole and effective brain tissue penetration of radiolabeled isavuconazole in healthy wistar rats [2, 32]. Isavuconazole also was shown to have similar brain tissue and CSF penetration, along with noninferior fungal killing efficacy to fluconazole in a rabbit model of *C. neoformans* meningoencephalitis [33]. Whether isavuconazole is noninferior to fluconazole in treating *C. gattii* meningoencephalitis in humans is unknown.

## Supplementary Data

Supplementary materials are available at *Open Forum Infectious Diseases* online. Consisting of data provided by the authors to benefit the reader, the posted materials are not copyedited and are the sole responsibility of the authors, so questions or comments should be addressed to the corresponding author.

ofz460_Suppl_Supplementary_Figures
