# Corrigendum

**DOI:** 10.1093/ofid/ofz542

**Published:** 2020-01-11

An error appeared in an article published in the issue of the journal [Corticosteroids for Posttransplant Immune Reconstitution Syndrome in *Cryptococcus gattii* Meningoencephalitis: Case Report and Literature Review.

Canfield GS, Henao-Martínez AF, Franco-Paredes C, Zhelnin K, Wilson ML, Shihadeh KC, Wyles D, Gardner EM. Open Forum Infect Dis. 2019 Oct 23;6(11):ofz460. doi: 10.1093/ofid/ofz460. eCollection 2019 Nov.].

In addition to the listed affiliations by all authors, CFP has a second affiliation: Hospital Infantil de Mexico, Federico Gomez, Mexico City, Mexico. The authors regret this omission.

