# Supplementary material for: Corticosteroids for Posttransplant Immune Reconstitution Syndrome in Cryptococcus gattii Meningoencephalitis: Case Report and Literature Review
Source: Open Forum Infect Dis. 2019 Oct 23;6(11):ofz460. doi: 10.1093/ofid/ofz460 (PMC6847472; doi:10.1093/ofid/ofz460)
Supplement: ofz460_Suppl_Supplementary_Figures [file ofz460_suppl_supplementary_figures.docx]

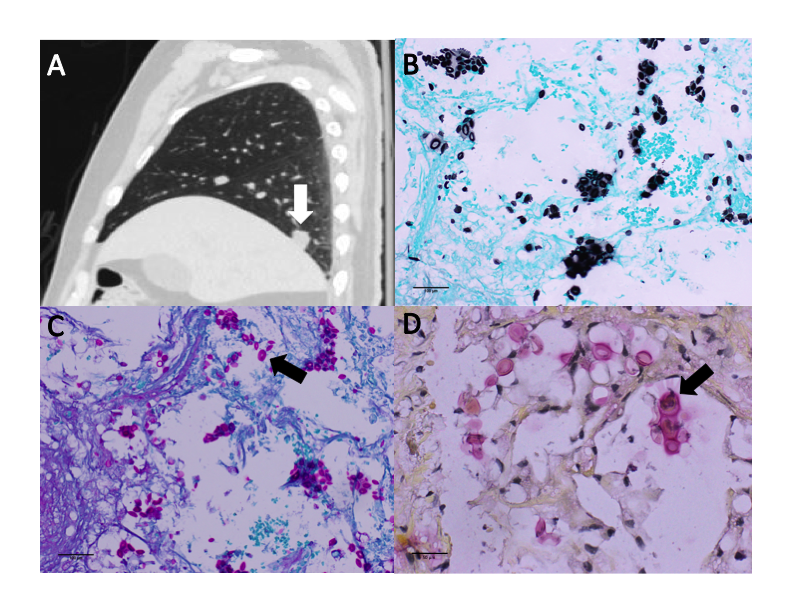


**Supplemental figure 1**: Computed tomography, sagittal section of the chest revealing pulmonary cryptococcomas in the middle and lower lobes of the right lung (A). The right lower lobe solid pulmonary nodule (white arrow, Figure 1A) was percutaneously biopsied and stained with Grocott Methenamine Silver (GMS; 200x magnification, Figure 1B), Periodic Acid Schiff (PAS, 200x magnification, Figure 1C), and Mucin stains (400x magnification, Figure 1D). Multiple yeast forms are observed with GMS, PAS, and mucin stain in the background of fibrotic tissue and debris. Capsule is demonstrated by PAS and mucin staining (black arrows, Figure 1C and 1D). Scale bars (lower left hand corner) in Figure 1B and 1C represent 100µm and Figure 1D represents 50µm.


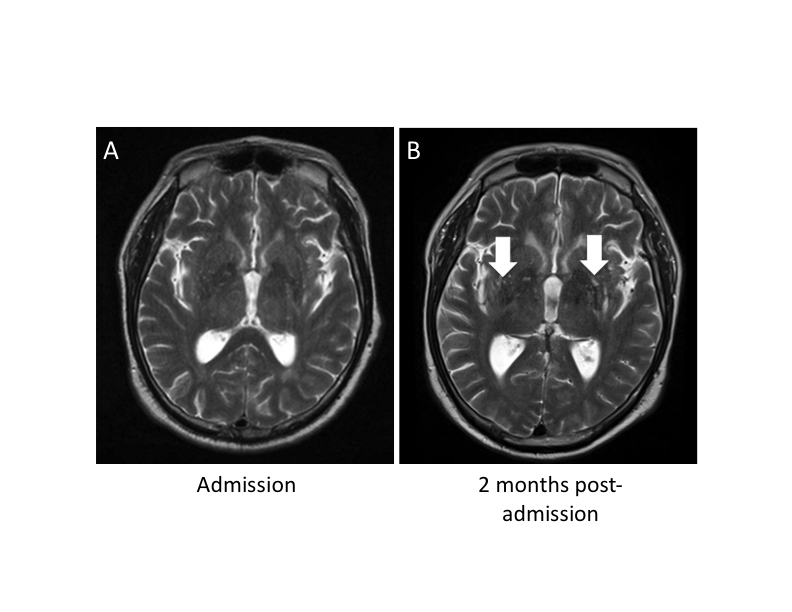


**Supplemental Figure 2**: T2 Axial MRI images on admission (A) and 2 months post admission (B). White arrows (panel B) indicate dilated Virchow-Robin (perivascular) spaces involving the putamen of the bilateral basal ganglia.
